# Predictors of postendoscopic retrograde cholangiopancreatography associated cholangitis: a retrospective cohort study

**DOI:** 10.3906/sag-2109-84

**Published:** 2022-01-23

**Authors:** Hasan YILMAZ, Burcu KOÇYİĞİT

**Affiliations:** 1 Division of Gastroenterology, Department of Internal Medicine, Faculty of Medicine, Kocaeli University, Kocaeli, Turkey; 2 Department of Internal Medicine, Faculty of Medicine, Kocaeli University, Kocaeli, Turkey

**Keywords:** Post-ERCP cholangitis, malignant biliary obstruction, ASA score, procedure duration

## Abstract

**Background/aim:**

Post-ERCP cholangitis (endoscopic retrograde cholangiopancreatography) and associated sepsis can be life-threatening. Despite the wealth of studies on post-ERCP pancreatitis risk factors, there is limited data on post-ERCP cholangitis. This study aimed to investigate the rates, predictors, and outcomes of post-ERCP cholangitis.

**Materials and methods:**

A retrospective review of 452 ERCP cases performed by a single endoscopist at a tertiary center between March 2019 and February 2021 was performed. Patient-related, organizational and periprocedural factors that could affect post-ERCP cholangitis were evaluated. Predictors of post-ERCP cholangitis were determined by multivariable analysis.

**Results:**

The post-ERCP cholangitis rate was 19.5%. Cholangiocarcinoma (OR 15.72, CI 2.43–101.55, p = 0.004), the American Society of Anesthesiologist Score (ASA) (OR 2.87, CI 1.14–7.21, p = 0.024), an increase in bilirubin after ERCP (OR 1.81 CI 1.01–3.22, p = 0.043), body mass index (OR 1.15, CI 1.00–1.33, p = 0.04) and procedure duration (OR 1.02, CI 1.00–1.05, p = 0.049) were predictors of post-ERCP cholangitis. Biliary stone extraction using a balloon was found to be protective against cholangitis (OR 0.18, CI 0.05–0.60, p = 0.005). Sepsis rate related to post-ERCP cholangitis was 2.4% and death 1%.

**Conclusion:**

Patients who undergo ERCP procedures due to malignant bile duct stenosis, have a high ASA score and BMI, and have a long procedure time should be paid attention and closely monitored. Further research is needed to determine whether measures aimed at the identified risk factors will reduce the incidence of post-ERCP.

## 1. Introduction

The endoscopic retrograde cholangiopancreatography (ERCP) procedure is widely used with considerable success in the treatment of bile duct and pancreatic pathologies in gastroenterological practice [1]. This effective procedure is the leading therapeutic tool for dealing with biliary stones, as well as with benign and malignant strictures [2]. With the widespread use of magnetic resonance cholangiopancreatography and endoscopic ultrasonography, the rate of diagnostic ERCP and ERCP-related complications have declined, while the preference for therapeutic ERCP has increased. However, life-threatening complications such as pancreatitis, cholangitis, bleeding and perforation, make this procedure more challenging in gastroenterological practice [3]. Post-ERCP pancreatitis rates and prevention methods have been extensively studied, and complication rates have been found to decrease through taking the appropriate measures. However, data on the factors that may predict post-ERCP cholangitis are limited, and further studies are needed on this subject.

Post-ERCP cholangitis is one of the most common complications observed after ERCP [4]. Cholangitis rates after ERCP vary between 1% and 7% [5,6] in the general population where all ERCP complications are reported, but this rate increases to 29.2% in patients who undergo ERCP and who have had a biliary stent inserted due to the indication of cholangiocarcinoma [7]. Post-ERCP cholangitis is the leading cause of the majority of septic complications after ERCP [8]. In a large multicenter, nationwide study, post-ERCP cholangitis was an independent predictor of post-ERCP sepsis. In this study, the rate of post-ERCP sepsis was 8.8% and related mortality was 7.5% [9]. To prevent such catastrophic clinical outcomes, it is necessary to know the rates of post-ERCP cholangitis, predict which patients are at high risk of developing post-ERCP cholangitis and take precautions for this patient group. According to the limited number of previous studies, age, prior history of ERCP, hilar obstruction [10], cancer diagnosis, multiple biliary stent insertion, and lower albumin levels [11] have been identified as independent factors for the development of post-ERCP cholangitis. However, compared to the more widely studied post-ERCP pancreatitis, there is a shortage of data related to factors that influence post-ERCP cholangitis.

This study aimed to ascertain post-ERCP cholangitis rates and describe the factors leading to cholangitis, in order to identify the interventions that can reduce post-ERCP cholangitis and sepsis rates in the future.

## 2. Methods

### 2.1 Design and settings

We reviewed ERCP procedures retrospectively to determine the rates and predictors of post-ERCP cholangitis in an inner-city tertiary health care facility between March 2019 and February 2021. All consecutive ERCP patients older than 18 years of age who gave written consent for the procedure were included in the study. Clinical histories, physical examination notes, laboratory values and ERCP reports of the patients were reviewed on the basis of the hospital’s electronic records. Excluded from the study were patients diagnosed with cholangitis before ERCP, patients who had been referred to our endoscopy unit for ERCP from another hospital and who were followed up at an external center after the procedure, patients who underwent cholangioscopy procedures but had inconsistent electronic medical records, as well as patients who, before the ERCP procedure, were already receiving antibiotics for some reason.

Over the ten years under examination, ERCP procedures at our hospital were performed by a total of thirteen different endoscopists, including five academic staff and eight fellows. Our ERCP unit was renovated two years ago and moved to a different location. Scope reprocessing procedures and logistics factors have changed. Because these factors may change the outcome of cholangitis, only data from an experienced endoscopist in the past two years was analyzed.

### 2.2 Definition of variables

Post-ERCP cholangitis served as the dependent variable, whereas the following variables were considered independent factors in post-ERCP cholangitis: sex, age, body mass index (BMI), hospitalization status, hospitalization duration (in days), comorbidities (hypertension, diabetes mellitus), number of the previous ERCPs, American Society of Anesthesiologists Score (ASA), tumor and stenosis localization of the biliary system, total procedure duration, ERCP cannulation technique, stone extraction technique (balloon or basket), and biliary stenting. Post-ERCP cholangitis is defined as abdominal pain, jaundice, and a body temperature of 38 degrees Celsius (°C) or above. Patients were accepted as post-ERCP cholangitis in the presence of the following findings in absence of any other infectious reason; leukocytosis >9000/μL, CRP > 3 mg/L, total bilirubin levels > upper limit of normal (ULN). The duration of the procedure was calculated retrospectively from the fluoroscopy and endoscopy records. The difference between the time of the first recorded image and the last was considered procedure time. The increase in bilirubin after the procedure was accepted as an elevation in the amount of bilirubin compared to the preprocedural levels. ASA scores were determined during preoperative evaluation at the anesthesiologist’s office before the ERCP appointment. ASA scores are as follows: I- a normal healthy patient, II- a patient with mild systemic disease, III- a patient with severe systemic disease, IV- a patient with severe systemic disease that is a constant threat to life, V- a patient who is not expected to survive without the operation, and VI- a declared brain-dead patient whose organs are being removed for donor purposes. Patients with ASA IV and above were intubated. Because of the higher periprocedural risk, the procedure was performed in the operating room close to the intensive care unit, instead of in the usual endoscopy suite.

### 2.3 ERCP procedure

ERCP procedures were performed under deep sedation, using propofol and midazolam. Heart rhythm, blood pressure, and oxygen saturation were closely monitored by the anesthesia team. After 6 h of observation following the ERCP procedure, patients without complaints and with stable vital signs were discharged and followed up on an outpatient basis. Prior to discharge, the patient and caregivers were verbally informed of the possible complications after ERCP. In case of any complaints, they were asked to inform the hospital via telephone or admit themselves to our hospital. The day after the procedure, all ERCP patients were followed up with a phone call. Patients who developed cholangitis in the long term and were admitted to a different hospital were followed up retrospectively using the e-nabız system for 30 days, and the results of the examination were evaluated. The e-nabız system is the national electronic medical record system through which doctors can access examinations performed all over Turkey.

### 2.4 Statistical analysis

All statistical analyses were performed using IBM SPSS Statistics 20.0 for Windows (IBM Corporation, Armonk, NY, USA). Kolmogorov–Smirnov and Shapiro–Wilk’s tests were used to assess the assumption of normality. Numeric variables were presented with mean ± standard deviation or median (25th–75th percentile). Categorical variables were summarized as counts (percentages). Comparisons of numeric variables between groups were carried out using the Mann–Whitney U test, since the normality assumption was not met. Association between two categorical variables was examined using the chi-square test. Logistic regression analysis was used to determine the factors that affected the outcome variable. All statistical analyses were carried out with 5% significance, and a two-sided p-value <0.05 was considered statistically significant.

### 2.5 Ethical considerations

This study protocol was reviewed and approved by the Kocaeli University Ethical Committee of Clinical Research (Identifier: GOKAEK-2021/13.15 Project number: 2021/219). The study was not funded by any organization. We conducted this research in accordance with the 1964 Helsinki Declarations.

## 3. Results

### 3.1 Patients and clinical characteristics

A total of 452 ERCP patients were initially identified during the 23-month study period, but only 296 patients were included in the analysis (Figure). Of these patients, 58 (19.5%) were diagnosed with post-ERCP cholangitis. One hundred and sixty-eight (56.8%) of the patients were men, the mean age of the study population was 60.64 ± 15.9 years, and 212 (71.6%) of the patients were inpatients (Table 1). One hundred and seventeen (40%) of the patients were naive for ERCP, while the rest of the patients experienced ERCP procedure one or more (Table 2). ERCP procedures lasted for a median of 21 min (IQR: 11.25–35). Patients with a diagnosis of cholangitis received inpatient treatment for a median of 5 days (IQR: 2.25–20.75). A total of 47 (81%) of the patients were diagnosed with cholangitis 48 h after the procedure. Post-ERCP sepsis developed in 7 patients (2.4%), and 3 of those patients died within 30 days after ERCP. The first who died was a 70-year-old male patient diagnosed with primary pancreatic head carcinoma, who had had a metal stent inserted by ERCP. He was found to be positive for COVID-19 on the 5th day after the procedure and died due to sepsis on the 9th day. The second patient, an 81-year-old female, had a previous history of diabetes mellitus type 2, hypertension, and breast carcinoma. She died as a result of septic shock on the 4th day after ERCP and after the insertion of a plastic stent, which was intended as a way of dealing with the periampullary mass. Finally, a biliary plastic stent was inserted by ERCP in an 87-year-old female patient with a diagnosis of pancreatic head carcinoma, and the patient who developed cholangitis and sepsis after the procedure died ten days afterwards.

### 3.2 Univariate analysis of factors associated with the cholangitis

According to our univariate analysis, patients with high BMI, an increased level of bilirubin after the procedure, long total procedure time, malignant disease, a high ASA score or plastic stent placement during ERCP, had a statistically significantly higher rate of post-ERCP cholangitis. Significantly less cholangitis was detected in patients who underwent a stone extraction with a balloon. There was no statistically significant difference in terms of sex, age, smoking, hypertension, diabetes mellitus type 2, antibiotic use before the procedure, cholecystectomy history, ERCP indication, sphincterotomy, number of placed stents, multiple ERCP history, and post-ERCP cholangitis (Table 3).

### 3.3 Multivariable analysis of factors associated with the cholangitis

The parameters that were found to be statistically significant in the univariate analysis were analyzed in the multivariable logistic regression model. The strongest independent predictor of post-ERCP cholangitis was cholangiocarcinoma (OR 15.72, CI 2.43–101.55, p = 0.004). In addition, ASA score, bilirubin increases after ERCP, BMI, and total procedure time were independent predictors of post-ERCP cholangitis. Biliary stone removal with the balloon was found to be a factor that protected against post-ERCP cholangitis. The association between metal or plastic stent placement, hospitalization duration, and cholangitis lost its significance in the multivariable analysis (Table 4).

## 4. Discussion

Post-ERCP cholangitis is a severe complication that can lead to sepsis and death if not treated promptly and appropriately. We retrospectively assessed post-ERCP cholangitis and determined post-ERCP cholangitis predictors for the additional targeted protective measures. We found that almost one-fifth of the patients examined developed cholangitis after ERCP. Our post-ERCP cholangitis rates were in the range of 1%–29.2% and comparable with the previous studies [7,12,13]. We found that the presence of cholangiocarcinoma, total procedure time, ASA score, bilirubin increases after ERCP, and BMI were independent predictors of post-ERCP cholangitis.

Our post-ERCP cholangitis rates are relatively high compared to the rates in those studies that examined general post-ERCP complications (ranged between 1% and 5%) [13,14]. In previous research, the definition of post-ERCP cholangitis was quite heterogeneous. For example, in one study, right upper quadrant pain, fever, leukocyte count > 12,000/μL, and CRP > 3 mg/L, ALT, and total bilirubin > ULN were considered acute cholangitis [15]. In that study, all patients were given prophylactic antibiotics before the procedure, and the highest rate of acute cholangitis was found to be 4.8%. In another study, fever of 37.8 °C and above, WBC > 9000/μL or <4000/μL, and CRP increase within seven days after ERCP, were regarded as indices of post-ERCP cholangitis, and post-ERCP cholangitis rates were calculated as 40.7% after plastic stent placement in patients with cholangiocarcinoma [16]. In our study, we accepted patients who had cholangitis in cases of leukocytosis, elevated total bilirubin, and CRP > ULN if there was no other cause of infection; we also looked for fever (37.8 °C), abdominal pain, and jaundice classical triad. Firstly, due to this broad definition, our post-ERCP cholangitis rates may have been high. Secondly, most of our study population consisted of cholangiocarcinoma and patients who received a biliary stent, so our cholangitis rate is comparable to that in studies whose subjects also experienced cholangiocarcinoma and stent-related cholangitis. Lastly, our study population consisted of tertiary university hospital patients. Thus, patients with more severe conditions may have been included. It is worth noting that our sepsis and mortality rate was low despite the high cholangitis rates. This suggests that post-ERCP cholangitis was recognized in a timely manner, and that adequate and critical treatment was provided.

The novel finding of our study was that ASA grade was an independent and strong predictor of post-ERCP cholangitis. Coton et al. concluded that poorer health conditions and ASA grades III–V were related to severe and fatal outcomes of ERCP [17]. Kwak et al. also concluded that ASA grades IV and V were related to overall post-ERCP complication. Additionally, ASA III was related to post-ERCP pancreatitis [18]. The ASA grade may reflect patients who are more vulnerable to ERCP complications, as it serves as a classification of a patient’s overall well-being. Therefore, one could expect advanced ASA grades to be linked to cholangitis. Our results revealed for the first time that ASA grade III was an independent predictor of post-ERCP cholangitis. In support of this, a moderate or severe illness which was previously indicated by the advanced ASA grade was associated with the surgical site infection after elective rectal cancer operation [19], shoulder arthroplasty [20], and prosthetic joint infections following the knee replacement [21]. For patients with an advanced ASA grade who plan to undergo the ERCP procedure, special care should be taken in terms of post-ERCP cholangitis, and these patients might be candidates for prophylactic antibiotic therapy.

We also found that BMI and the total procedure time for cholangiocarcinoma were independent risk factors for post-ERCP cholangitis. Our results confirmed that cholangiocarcinoma was an independent risk factor leading to post-ERCP cholangitis [10,22]. In case of hilar obstruction, it may not always be possible to provide biliary drainage, or this drainage may be insufficient due to stricture. In addition, even if a stent can be placed proximal to the stenosis, early stent occlusion is most common at hilar localization [23], and post-ERCP cholangitis may develop subsequent insufficient drainage. As a result of a complete or partial obstruction in the biliary system due to cholangiocarcinoma, the biliary pressure rises and, accordingly, cannulation becomes difficult during ERCP. In addition, the transition to the proximal of stenosis is challenging, and the duration of the procedure is prolonged. Prolonged procedure time can cause mucosal damage, and bacterial endotoxins can easily penetrate the deteriorated blood-biliary to the mucosa to enter the systemic circulation and cause bacteremia. In line with a previous study, we found that prolonged procedure time increases the risk of cholangitis [24]. Additionally, the possibility of developing cholangitis rises with increasing BMI. It is known that patients with a high BMI are more vulnerable to hospital and community-acquired infections as a result of impaired immune function [25]. Patients with a high BMI tend to have metabolic disorders, are vulnerable to infections, and are at greater risk for nosocomial infections, especially after the interventional procedure.

Taking appropriate measures for the defined risk factors associated with post-ERCP cholangitis could result in a decrease in the cholangitis rate and enhance positive outcomes. Kim et al. compared the effectiveness of preprocedural intravenous moxifloxacin and ceftriaxone with patients undergoing ERCP. Post-ERCP cholangitis occurred in only 2.3% of the moxifloxacin group and 4.8% of the ceftriaxone group. There was no statistically significant difference between groups [15]. According to the American Society for Gastrointestinal Endoscopy (ASGE) guideline recommendations, antibiotic usage is not routinely recommended before ERCP, but one should consider initiating antibiotics for patients at high risks, like suspicion of incomplete biliary drainage [26]. On the one hand, oral care, washing the duodenum lumen, and scope working channel could prevent microorganism inoculation in the bile duct and consequently reduce the cholangitis rate [27]. However, adding antibiotics to the contrast dye did not change the post-ERCP cholangitis incidence rate [28]. These suggested interventions need confirmation with prospective, more extensive studies.

Using a retrospective cohort was one of the main limitations of our study. This study design comes with the possibility of selection bias. Another limitation was that the studied population consisted of a relatively small number of patients. Therefore, we may have underestimated the extent of post-ERCP cholangitis-related factors and missed small statistical changes. Given that we conducted this study at a tertiary healthcare facility, it is possible that we included patients in more severe conditions. Consequently, the generalizability of our results to community-based healthcare facilities remains unclear.

Despite the limitations, we analyzed only one endoscopist’s ERCPs to eliminate the heterogeneity that may arise from the experience of different endoscopists. We also use renewed ERCP scopes and fixed scope reprocessing procedures such as instrument washing and drying units to rule out potential confounds according to international guidelines [29]. Lastly, patients with unreliable and inconsistent data in our hospital’s electronic records were meticulously eliminated from the study.

In conclusion, patients with high ASA and BMI scores, prolonged total procedure time, and bilirubin increase after the procedure were at risk for developing post-ERCP cholangitis. Successful removal of the biliary stone with the balloon was protective against cholangitis. Our research was exploratory. Thus, future studies are needed to evaluate whether measures against these risk factors will reduce cholangitis rates.

## Figures and Tables

**Figure:**
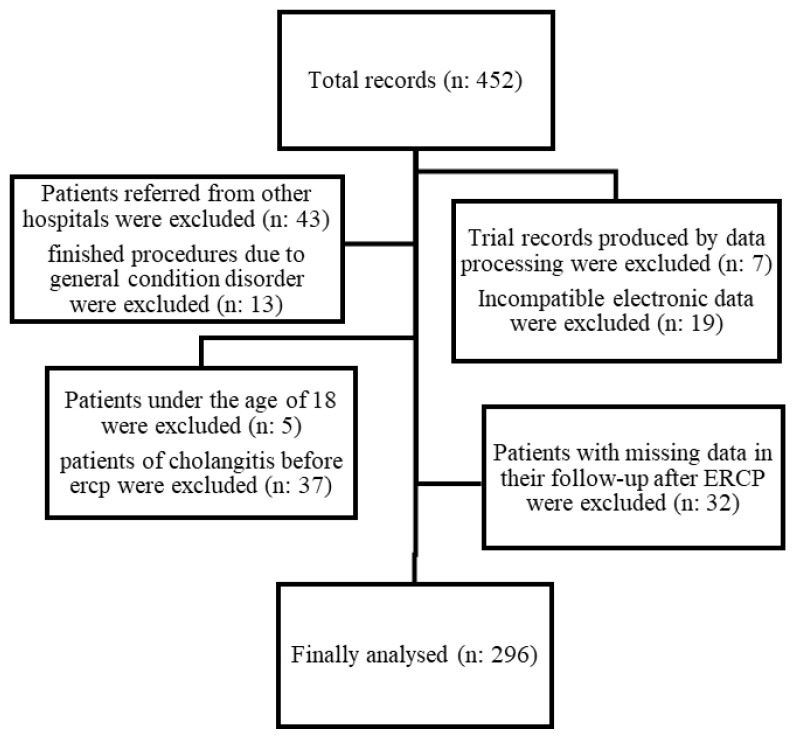
Flowchart of the study population.

**Table 1:** Baseline characteristics of patients (n = 296).

	N	(%)
Sex (male)	168	56.8
Age*	60.6 ± 15.9	(18–94) **
BMI*	26 ± 4.3	(17–46) **
Hospitalization (days)*	5 ± 11	(1–60) **
Smoking	59	19.9
Malignancy	86	29.1
Cholecystectomy	72	24.3
**Hospital stays**		
In-patients	212	71.6
Outpatients	84	28.4
**Comorbidities**		
Hypertension	114	38.5
Diabetes mellitus	95	32.1
**Number of ERCP**		
None	117	39.5
Once or twice	127	42.9
Three time or more	52	17.6
**ASA scores**		
ASA I	55	18.6
ASA II	147	49.7
ASA III	82	27.7
ASA IV	12	4.1

ASA: American Society of Anesthesiologists, ERCP: Endoscopic Retrograde Cholangiopancreatography, BMI: Body Mass Index kg/m^2^,

* (mean ± SD),

** minimum-maxiımum values.

**Table 2:** Baseline procedure characteristics (n = 296).

	N	(%)
**ERCP indications**		
Periampullary Tm	6	2
Cholangiocarcinoma	62	21.0
Pancreas Ca	45	15.2
Choledocholithiasis	126	42.6
Stent revision	43	14.5
Hilar metastasis	14	4.7
**Techniques of ERCP**		
Sphincterotomy	225	76
Precut papillotomy	37	12.5
Extraction balloon	192	64.9
Basket	50	16.9
Brush cytology	18	6.1
**Number of biliary stents**		
No stent	119	40.2
One stent	140	47.3
Two stent	37	12.5
**Stent type**		
Plastic stent	145	81.9
Metallic stent	32	18.1
CBD diameter (mm)*	12.4 ± 5.2	(5–30) **
Procedure duration (min)*	24.5 ± 17.9	(2–130) **

ERCP: Endoscopic Retrograde Cholangiopancreatography, Tm: Tumor, Ca: Carcinoma, CBD: Common Bile Duct, min: minutes, mm: millimeter,

* mean ± SD,

** minimum-maxiımum values.

**Table 3:** Comparison of risk factors for post-ERCP cholangitis.

	Patients without cholangitis (n = 238)	Patients with cholangitis (n = 58)	p value
	N / (%)	N / (%)	
**Sex**		0.981^a^	
Male	135 (56.7)	33 (56.9)	
Female	103 (43.3)	25 (43.1)	
Age *	60.4 ± 16 (18–94) **	61.3 ± 15.9 (24–92) **	0.639 b
BMI*	25.7 ± 4.3 (17–46) **	27 ± 3.9 (21–37) **	**0.013** b
In-patients	161 (67.6)	51 (87.6)	**0.004** a
Hospitalization (days)*	4 ± 8.6 (0–60) **	11 ± 15.8 (0–60) **	**0.001** b
Smoking	45 (18.9)	14 (24.1)	0.477 a
Hypertension	95 (39.9)	19 (32.8)	0.393 a
Diabetes mellitus	76 (31.9)	19 (32.8)	1.000 a
Malignancy	61 (25.6)	25 (43.1)	**0.014** a
Cholecystectomy	59 (24.8)	13 (22.4)	0.836 a
Post-ERCP fever	22 (9.2)	31 (53.4)	**<0.001** a
Increase in bilirubin (mg/dL) *	0.5 ± 0.8 (0–6) **	1.8 ± 2.7 (0–15) **	**<0.001** b
CBD diameter (mm)*	12.7 ± 5.4 (5–30) **	11.2 ± 4.2 (6–20) **	0.097 b
Procedure duration (min)*	22.7 ± 16 (2–80) **	32 ± 23 (3–130) **	**0.001** b
Sphincterotomy	177 (74.4)	48 (82.8)	0.242 a
Precut papillotomy	31 (13)	6 (10.3)	0.740 a
Extraction balloon	172 (72.3)	20 (34.5)	**0.001** a
Basket	42 (17.6)	8 (13.8)	0.612 a
**Number of biliary stents**		0.060 a	
No stent	103 (43.3)	16 (27.6)	
One stent	105 (44.1)	35 (60.3)	
Two stent	30 (12.6)	7 (12.1)	
**Stent type**		**0.020** a	
Plastic stent	107 (45.5)	38 (65.5)	
Metallic stent	28 (11.8)	4 (6.9)	
**Number of ERCP**		0.962 a	
None	95 (39.9)	22 (37.9)	
Once or twice	101 (42.4)	26 (44.8)	
Three-time or more	42 (17.6)	10 (17.2)	
**ASA scores**		**0.002** a	
ASA I	52 (21.8)	3 (5.2)	
ASA II	121 (50.8)	26 (44.8)	
ASA III	58 (24.4)	24 (41.4)	
ASA IV	7 (2.9)	5 (8.6)	
**Tumor localization**			
Pancreatic head	43 (18.1)	16 (27.6)	0.149 a
CBD	16 (6.7)	21 (36.2)	**0.001** a
Periampullar	8 (3.4)	7 (12.1)	**0.014** a

ASA: American Society of Anesthesiologists. ERCP: Endoscopic Retrograde Cholangiopancreatography, CBD: Common Bile Duct, BMI: Body Mass Index kg/m^2^, min: minutes, mg/dL: milligrams per decilitre, mm: millimeter,

* mean ± SD.

** minimum-maxiımum values,

a Chi-squared test,

b Mann–Whitney U test.

**Table 4:** Multivariable logistic regression analysis of risk factors for post-ERCP cholangitis.

			95% CI	
	p value	OR	Lower	Upper
Cholangiocarcinoma	0.004	15.722	2.434	101.552
ASA score	0.024	2.875	1.146	7.214
Increase in bilirubin	0.043	1.813	1.018	3.227
BMI	0.048	1.154	1.001	1.330
Procedure duration (min)	0.049	1.027	1.000	1.055
Hospitalization (days)	0.428	1.017	0.976	1.060
**Stent type**				
None	0.45	1.0 (reference)		
Plastic stent	0.25	1.952	0.617	6.173
Metallic stent	0.85	0.788	0.093	6.666

BMI: Body Mass Index kg/m^2^, CI: Confidence Interval, OR: Odds Ratio, min: minutes.
